# Effectiveness of creative story therapy for dementia: a systematic review and meta-analysis

**DOI:** 10.1186/s40001-023-01337-7

**Published:** 2023-09-14

**Authors:** Jinlong Ma, Qian Wang, Yanmei Lang, Shi Lv, Yuzhen Xu, Baojian Wei

**Affiliations:** 1 School of Nursing, Yanbian University, Yanji, Jilin, China; 2 Department of Central Laboratory, The Affiliated Taian City Central Hospital of Qingdao University, Taian, Shandong China; 3 Department of Rehabilitation, The Second Affiliated Hospital of Shandong First Medical University, Taian, Shandong China; 4 School of Nursing, Shandong First Medical University & Shandong Academy of Medical Sciences, Taian, Shandong China

**Keywords:** Dementia, Alzheimer’s disease, Creative story, Meta-analysis, Clinical trials

## Abstract

**Objective:**

To conduct a meta-analysis of the effectiveness of creative story therapy versus routine nursing alone for the treatment of dementia.

**Methods:**

We manually searched PubMed, Embase, Cochrane Library, Web of Science, China National Knowledge Infrastructure (CNKI), China VIP Database (VIP), China Biomedical Literature Database (CBM), and Wanfang Data up to May 2023. Randomized controlled trials (RCTs) of creative story therapy combined with routine nursing versus routine nursing for the treatment of dementia were included.

**Results:**

A total of 597 participants were enrolled in the 9 RCTs. Among them, 296 were in the creative story therapy group, and 301 were in the routine nursing group. We found statistically significant effects of creative story therapy combined with routine nursing on cognitive function [standardized mean difference (SMD) = 0.99, 95% CI 0.57, 1.41, *P* < 0.00001], CSDD score [mean difference (MD) =  − 1.71, 95% CI − 3.27, − 0.14, *P* < 0.00001], quality of life [SMD = 0.97, 95% CI 0.04, 1.90, *P* = 0.04], and social communication [MD = 0.46, 95% CI 0.17, 0.74, *P* < 0.00001] between the creative story therapy group and routine nursing groups; no significant difference in change in basic needs communication [MD = 0.09, 95% CI − 0.58, 0.76, *P* < 0.00001].

**Conclusion:**

This meta-analysis shows that creative story therapy combined with routine nursing has significant effectiveness in improving cognitive function and depression in people with dementia. More high-quality RCTs are required to validate these results.

**Supplementary Information:**

The online version contains supplementary material available at 10.1186/s40001-023-01337-7.

## Introduction

Dementia refers to a group of degenerative diseases of the central nervous system, characterized primarily by cognitive impairments [1]. According to the latest data from the World Health Organization (2023), there are approximately 55 million people with dementia (PWD) worldwide [2]. With global population aging and increased life expectancy, it is projected that the number of PWD will reach 130 million by 2050, posing significant challenges to global healthcare systems [3]. Studies on the cost of dementia diseases have shown that the annual cost of dementia care is approximately $30,554, with long-term home care being the major cost driver, and the cost of nursing will increase dramatically, with the deterioration of dementia.[4, 5]

While the use of anti-dementia drugs can partially improve dementia symptoms, their therapeutic effects are limited, only able to control the progression of symptoms, and may come with some adverse reactions, along with the burden of long-term high costs [6]. In comparison, non-pharmacological treatments offer higher safety and acceptability [7]. Non-pharmacological approaches such as music therapy and art therapy have numerous advantages in improving the behavior and psychological symptoms of dementia patients, enhancing cognitive functions, promoting well-being, and reducing caregiver burden [8–10]. Recently, person-centered care (PCC) has gained increasing attention in dementia care [11, 12]. A study indicated that PCC [13], as a standard for providing long-term services and supports (LTSS) to dementia patients, can be an effective approach in preventing and managing dementia behaviors and psychological symptoms. Creative story therapy is one form of engagement in PCC, simulating genuine person-centered care [14]. Timeslips, created by Anne Basting, the director of the University of Wisconsin's Center on Age and Community, is an improvisational storytelling process and a typical form of creative story therapy [15]. In the Timeslips care process, patients typically sit comfortably in chairs around a facilitator, who guides them in telling stories related to images and other prompts, providing appropriate guidance and adjustments based on each patient's personality and characteristics, encouraging their participation in storytelling and sharing. The facilitator does not correct the content of the patients' stories, but creates various conditions for their engagement and repetition of the stories to facilitate communication and sharing among the patients [16]. Various theoretical perspectives support the benefits of creative story therapy, such as cognitive theory, relationship-centered care models, generativity theory, and social-emotional selectivity theory [17]. While these theories differ in content and application, they all emphasize the importance of cognitive factors, which are key to creative story therapy. Creative story therapy can empower dementia patients with memory and functional impairments to express themselves through imagination, thereby preserving their social roles and maintaining their individuality [18, 19]. This intervention process has little restriction on patients' cognitive function, especially memory ability, instead, it can be achieved through their existing capabilities [20]. Recent studies have shown that creative story therapy can help maintain and improve cognitive functions in dementia and mild cognitive impairment patients [17, 21]. A mixed-methods study also found the benefits of creative story therapy for dementia patients at various stages [22]. However, the effectiveness of creative story therapy has not been systematically evaluated, and thus, this study aims to meta-analyze its effectiveness.

## Methods

This meta-analysis was conducted according to the Preferred Reporting Items for Systematic Reviews and Meta-Analyses (PRISMA) guidelines. The systematic review methodology employed in this study has been registered on the International Platform of Registered Systematic Review and Meta-analysis Protocols (INPLASY), and the registration number is INPLASY202380041.

### Search strategy

A comprehensive scoping search was conducted to gather research published until May 11, 2023. Two independent authors (WBJ and JLM) searched the PubMed, Embase, Cochrane Library, Web of Science, China National Knowledge Infrastructure (CNKI), China VIP Database (VIP), China Biomedical Literature Database (CBM), and Wanfang Database. To ensure the effectiveness of the search, a combination of MeSH terms and relevant keywords was employed. The search terms for this review were ("dementia" or "Alzheimer's disease" or "dementia *" or "Alzheimer's disease *" or "senile dementia") and ("creativity" or "creative expression *" or "creative expression therapy" or "timeslips"). The retrieval strategy was jointly determined by two evaluators and involved a manual search of references in selected studies to identify articles that fulfilled the predetermined criteria.

### Qualifications

The inclusion criteria for this review are determined by the "PICOS" principle. The target population consists of individuals aged 60 and above, who were diagnosed with dementia according to the established diagnostic criteria: internationally accepted diagnostic criteria.

DSM-IV-R (Diagnostic and Statistical Manual of Mental Disorder, 4th edition, Revised) and ICD-10 (International Classification of Diseases, Tenth Revision). The intervention focuses on the experimental group receiving creative story therapy, specifically storytelling intervention, while the control group receives routine nursing intervention. The primary outcome of interest centers around overall cognitive function, which can be assessed through standardized scales such as the Mini Mental State Examination (MMSE), the Montreal Cognitive Assessment (MoCA), and the Alzheimer’s Disease Assessment Scale Cognitive Subscale (ADAS-cog). Secondary outcomes encompass depression, which can be evaluated using the Kangnai Depression Scale, and quality of life, which can be assessed using validated and standardized quality of life-specific scales for dementia patients, such as the Alzheimer's Disease Quality of Life Scale (QOL-AD). Communication skills can be evaluated through the Functional Assessment of Communication Skills (SFACS). Moreover, the included studies must be randomized controlled trials published in either Chinese or English. On the other hand, the exclusion criteria encompass reviews, qualitative research, conference abstracts, and experimental protocols. Additionally, original research articles without full-text availability or missing primary outcome data were excluded. Repeatedly published articles by the same authors or teams were also excluded. Furthermore, studies with low-quality literature evaluations are not considered in this review.

### Research selection and data extraction

The collected research was imported into the EndNoteX9.1 software. Duplicate studies were promptly identified and removed. The title and abstract of each article are then carefully assessed, allowing the reviewers to select studies that meet the predetermined inclusion criteria. To maintain accuracy and consistency, two independent reviewers, JLM and YML, screened and extracted the literature. They conduct thorough cross-checking to ensure the reliability of the selected studies. In case of any differences or disagreements, JLM and YML engaged in thoughtful discussions to reach a consensus. Alternatively, if needed, they seek advice from a third-party expert to resolve any remaining discrepancies. The following characteristics were extracted: author information, publication year, patient age, sample size, details of the intervention measures, duration of the intervention, and assessment scales.

### Quality assessment

Two evaluators, WBJ and JLM, independently employed the esteemed Cochrane Risk Bias Assessment Tool [23]. This tool, specifically designed for randomized controlled studies, comprises seven essential questions that comprehensively evaluate various aspects of study quality. These questions encompass critical domains, including random sequence generation, allocation concealment, blinding of implementation, blinding of outcome assessment, completeness of outcome data, selective outcome reporting, and potential biases. Each item is carefully classified as either low risk, unclear, or high risk, indicating the level of potential bias associated with the study. To ensure accuracy and consistency, any discrepancies or differences in evaluation are meticulously addressed. In such cases, the Third Reviewer, XYZ, serves as an impartial mediator, providing invaluable expertise to resolve any remaining disagreements and guide the final assessment.

### Statistical analysis

We used two software tools, RevMan 5.4 and Stata software (version 14), to perform comprehensive statistical analysis. All the outcome indicators examined in this meta-analysis consist of continuous variables. Depending on the consistency of the evaluation tools employed across studies, we will utilize different effect indicators. Specifically, when the evaluation tools are consistent, we will employ the mean difference (MD) as the effect indicator. Conversely, when variations in evaluation tools are inconsistent, we will employ the standardized mean difference (SMD) as the effect indicator. To determine the magnitude of the effect size, we will utilize Cohen's criterion. According to Cohen's criterion, effect sizes between SMD (or MD) ≥ 0.20 and < 0.50 are considered small, SMD (or MD) ≥ 0.50 and < 0.8 are considered moderate, and SMD (or MD) ≥ 0.8 are considered large, thereby offering a comprehensive understanding of the impact.

Heterogeneity among the included studies was assessed through the I^2^ statistic and Chi-square test statistics, and a random effects model was used in consideration of inevitable heterogeneity. Subgroup analysis was conducted to further investigate the potential sources of heterogeneity and sensitivity analysis was conducted to assess the impact of individual studies on the overall findings and the stability of the results. Additionally, to evaluate the potential publication bias, funnel plots will be utilized, providing a visual representation of the distribution of studies and their corresponding effect sizes. Furthermore, to detect and quantify any publication bias present, we will employ both funnel plots and Egger's test, a widely recognized method for assessing publication bias [24].

## Result

### Literature search

**Fig. 1:**
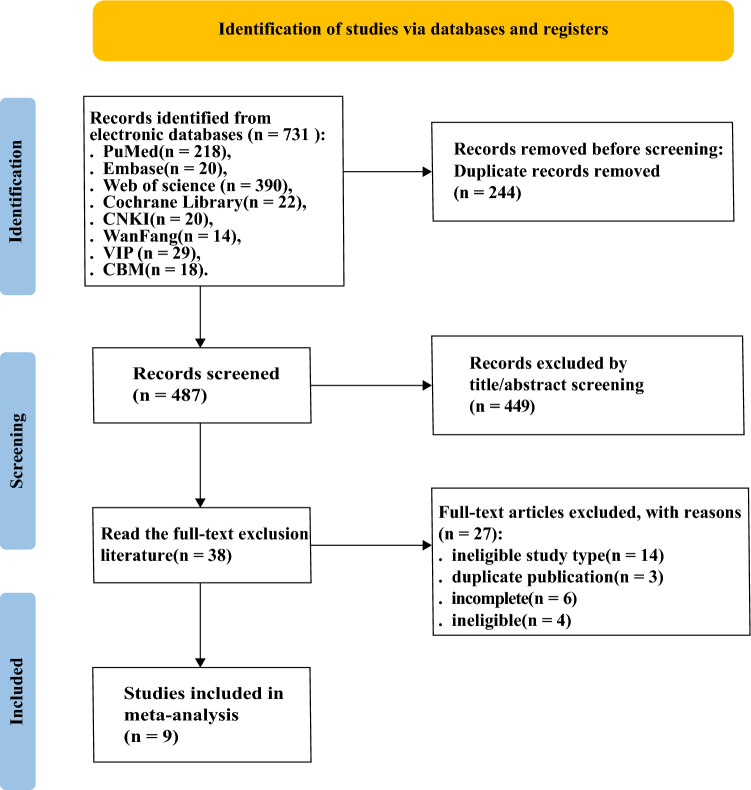
PRISMA flow diagram

Figure 1 shows the search strategy. In our literature search, a total of 731 articles relevant to our study were discovered. We removed 244 duplicate articles, leaving us with 487 unique and relevant articles to delve into. After a thorough evaluation of these articles against our eligibility criteria, we found that 9 studies were proved to be fitting for our meta-analysis [25–33].

### Study characteristics

Table 1 presents the basic characteristics of the nine included studies which were conducted ranging from 2010 to 2021, with an enrollment of 597 participants. Within this cohort, 296 participants were in the creative story therapy group, and 301 participants constituted the routine care group. The sample sizes within the studies varied from 20 to 112 cases. All studies reported the mean and SD of age for participants. The duration that the studies lasted ranged from 4 to 8 weeks. The studies reported different outcomes of interest, with seven studies exploring changes in cognitive function, five studies focusing on the Cornell Scale for Depression in Dementia (CSDD), four studies delving into quality of life, and two studies investigating the changes in communication skills before and after the patient intervention.

**Table 1:** Characteristics of the included studies

First author, (publication year)	Country	Diagnosis	Experimental Group *N*, age (mean ± SD) on baseline	Routine care Group N, age (mean ± SD) on baseline	Control intervention and duration/intensity	Outcomes measured:	Interventionist
Cai, 2019 [25]	China	Mild and moderate dementia	*N* = 20, Age = 68.45** ± **3.73	*N* = 20, Age = 69.85** ± **4.11	*Type of participation: Group ranging from 6-10 person* *Frequency:* 1 weekly *Duration:* 40–60-min *Length*: 7 weeks	Cognitive functioning CSDD	Nurse therapeutist
Fan, 2021[26]	China	Alzheimer disease	*N* = 56, Age = 69.83** ± **5.62	*N* = 56, Age = 69.44** ± **5.39	*Type of participation: Group ranging from 6-10 person* *Frequency:* 1 weekly *Duration:* 40–60-min *Length*: 6 weeks	Cognitive functioning	Nurse (host, recorder, site staff)
Lin, 2019 [27]	China	Dementia 11 ≤ MMSE < 17	*N* = 43, Age = 85.3** ± **5.89	*N* = 48, Age = 84.6** ± **8.11	*Type of participation:* *Group ranging from 6–7 person* *Frequency:* 2 weekly *Duration: 1 h Length:* 6 weeks	Cognitive functioning CSDD communication skills QOL-AD	Psychotherapists Facilitators, video recorder
Phillips, 2010 [28]	America	Dementia 11 ≤ MMSE < 24	*N* = 28, Age = 83.42** ± **8.0	*N* = 28, Age = 85.75** ± **6.2	*Type of participation:* *Group ranging from 6–12 person* *Frequency:* 2 weekly *Duration: 1 h Length:* 6 weeks	CSDD QOL-AD communication skills	first author study nurse facilitators
Shen, 2021 [29]	China	Mild dementia 211 ≤ MMSE < 26	*N* = 40, Age = 73.12** ± **7.3	*N* = 40, Age = 73.1** ± **7.49	*Type of participation:* *Group ranging from 6-8 person* *Frequency: 2* weekly *Duration: 1 h Length: 6* weeks	Cognitive functioning	Nurse Graduate students doctor
Wang, 2013 [30]	China	Dementia MMSE > 10	*N* = 29, Age = 83.0** ± **6.9	*N* = 29*,* Age = 82.4** ± **6.84	*Type of participation:* *1-to-1 intervention alone* *Frequency: 1 weekly* *Duration:* 45-min *Length: 8* weeks	Cognitive functioning CSDD DQOL	Nurse
Wang, 2020 [31]	China	Alzheimer disease	*N* = 38, Age ≥ 60	*N* = 38, Age ≥ 60	*Type of participation:* *Group ranging from 6–7 person* *Frequency: 1* weekly *Length: 4* weeks	Cognitive functioning	Nurses
Xia, 2020 [32]	China	Alzheimer disease	*N* = 32, Age ≥ 60	*N* = 32, Age ≥ 60	*Type of participation:* *Group ranging from 6–7 person* *Frequency: 1* weekly *Length: 4* weeks	Cognitive functioning	Nurses
Ye, 2019 [33]	China Taiwan	Mild and moderate dementia	*N* = 10, Age = 83.70** ± **3.8	*N* = 10, Age = 88.0** ± **4.42	*Type of participation:* *Group* *Frequency: 1* weekly *Duration: 1 h Length: 6* weeks	CSDD QOL	Nurses, social workers, care and service staff

### Methodology quality

**Fig. 2:**
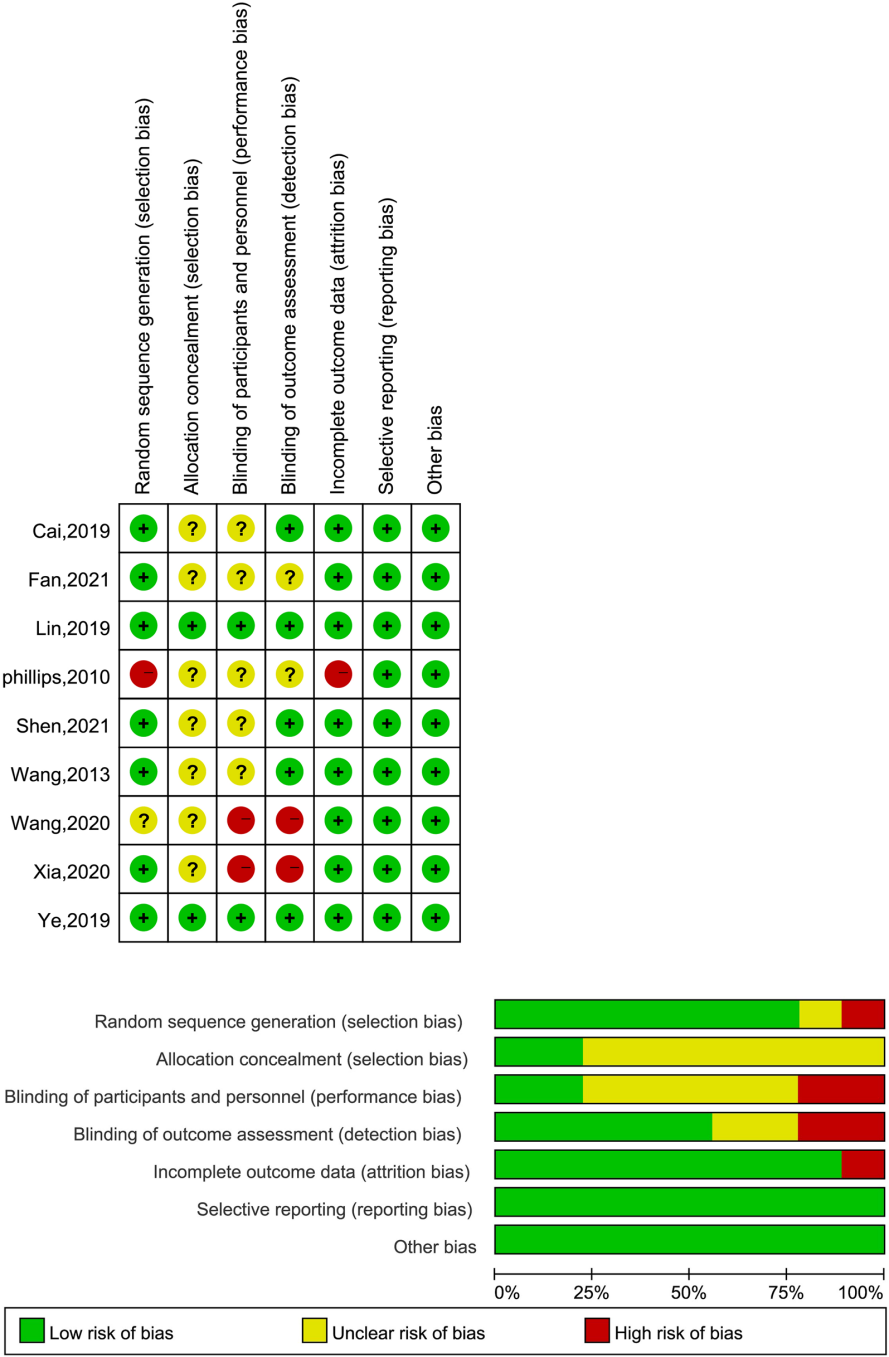
Risk-of-bias

In terms of random sequence generation, 6 studies were determined to be at low risk of bias while 3 studies were judged to be unclear. In regard to allocation concealment, only 2 studies were judged to have a low risk of bias, and the other trials were judged to have an unclear risk of bias. Blinding study subjects proved challenging due to ethical considerations. For blinding of personnel, only 2 studies provided detailed information regarding their blinding methods during intervention implementation, 5 studies were judged to be at an unclear risk and 2 studies were judged to be high risk primarily due to insufficient disclosure of specific intervention details. Three studies were considered as high risk on the outcome measures. All studies reported baseline comparability without selective reporting and other high risks of bias. For a comprehensive overview of the assessed risk of bias, please refer to Fig. 2 and Additional file 1: Table S1.

## Meta-analysis results

### Cognitive function

**Fig. 3:**
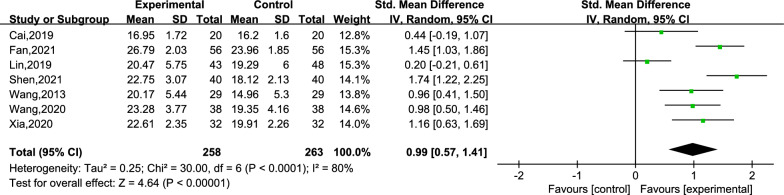
Meta-analysis of cognitive function

**Fig. 4:**
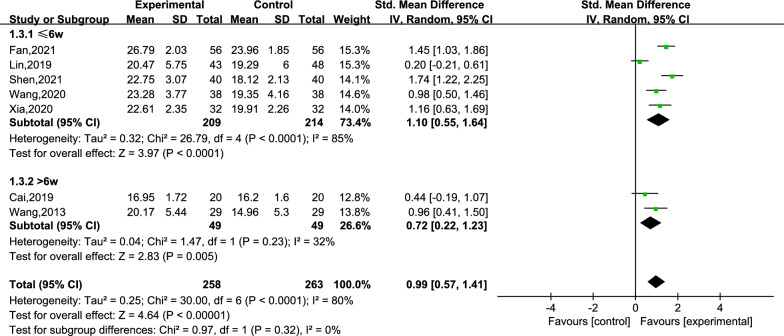
Subgroup analysis by intervention cycle of cognitive function

**Fig. 5:**
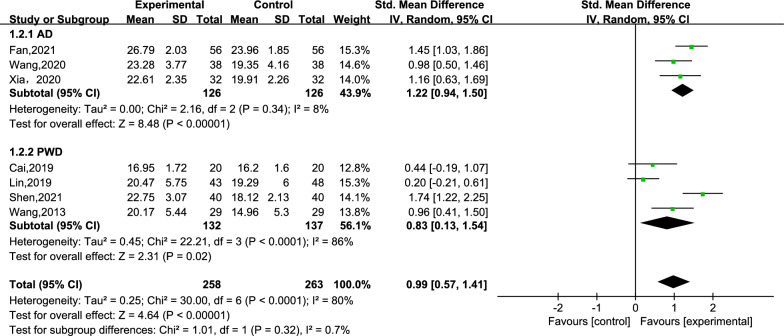
Subgroup analysis by types of dementia of cognitive function

An ensemble of 7 studies reported the scores for cognitive function [25–27, 29–32]. Within the 7 studies, a total of 521 subjects were divided into the creative story therapy group (258 subjects) and a routine care group (263 subjects). The meta-analysis results demonstrated that the creative story therapy group had significantly higher scores than the routine care group (SMD = 0.99, 95% CI 0.57, 1.41, *P* < 0.00001, *I*^2^ = 80%) (Fig. 3). Then, we conducted a subgroup analysis according to the intervention period (subgroups: intervention period ≤ 6 weeks and intervention period > 6 weeks). The heterogeneity within the two subgroups was *I*^2^ = 85% and 32%, respectively (Fig. 4), while the inter-subgroup heterogeneity was not very evident (*I*^2^ = 0%). The dementia type can be divided into subgroup AD and subgroup PWD (Fig. 5), and the heterogeneity within the two subgroups was *I*^2^ = 8% and 86%, respectively, and the inter-subgroup heterogeneity was not evident (*I*^2^ = 0.7%). The estimated SMDs were 1.22 and 0.83. We then performed a sensitivity analysis by changing the effect model and eliminating the literature one-by-one method, which showed that deleting any literature had little effect on the combined SMD values, that is, the results of the pooled result were reliable (refer to Additional file 2: Table S2).

**Fig. 6:**
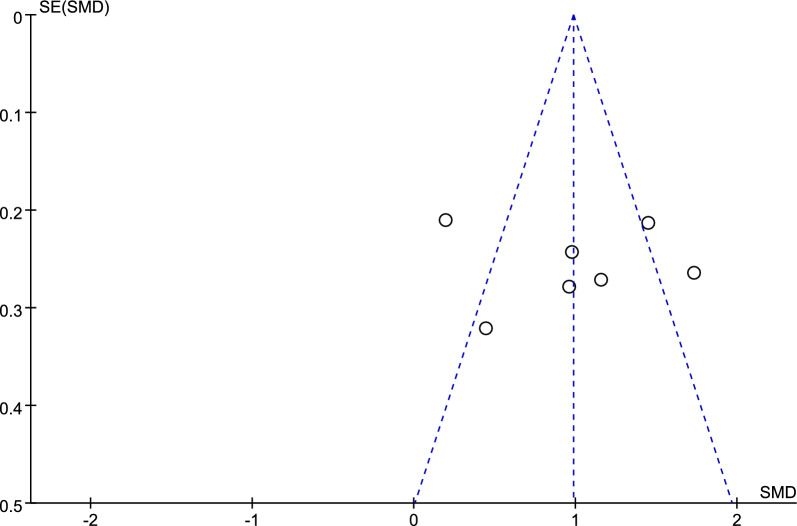
Funnel plot of cognitive function

Figure 6 shows a funnel plot of the cognitive function, and no obvious publication bias was observed according to the funnel plot and Eggers’ test (*p* = 0.872, refer to Additional file 2: Table S3).

### CSDD

**Fig. 7:**
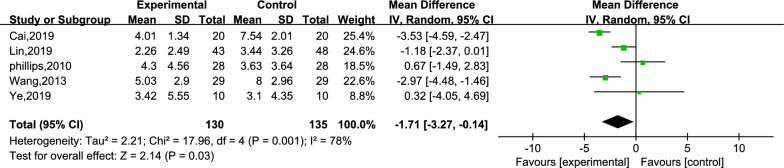
Meta-analysis of CSDD

**Fig. 8:**
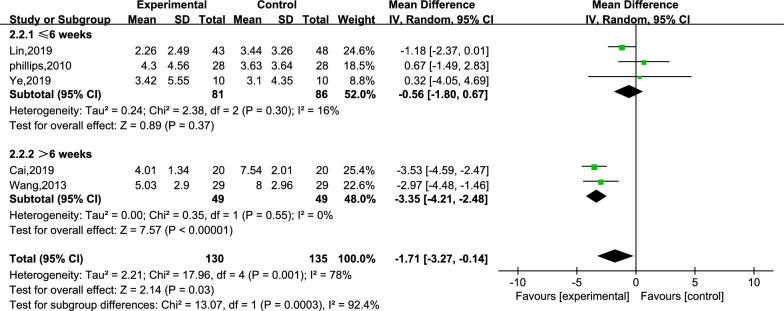
Subgroup analysis by intervention cycle of CSDD

Five studies reported the scores for cognitive function.[25, 27, 28, 30, 33] A total of 265 subjects, with 130 subjects in the creative story therapy group and 135 subjects in the routine care group, were included in these studies. The meta-analysis results revealed that the creative story therapy group had significantly lower scores than the routine care group (MD =  − 1.71, 95% CI − 3.27, − 0.14, *P* < 0.00001, *I*^2^ = 78%, Fig. 7). Then, we conducted a subgroup analysis, and the intervention period was divided into subgroups ≤ 6 weeks and subgroups > 6 weeks (Fig. 8), and the heterogeneity within the two subgroups was, respectively, not too evident (*I*^2^ = 16% and 0%), but the inter-subgroup heterogeneity was evident (*I*^2^ = 92.4%). The estimated MDs were − 0.56 and − 3.35. We then performed a sensitivity analysis by changing the effect model and eliminating the literature one-by-one method. Both subtotal and overall effect sizes were still steady with reduced heterogeneity after the two studies [27, 28] (refer to Additional file 1: Table S2) were removed.

**Fig. 9:**
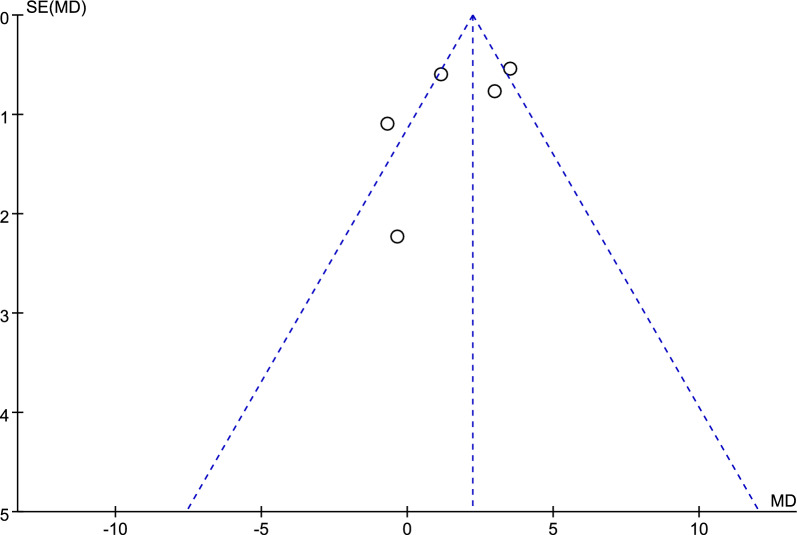
Funnel plot of CSDD

Figure 9 shows a funnel plot of the CSDD, and no obvious publication bias was observed according to Eggers’ test (*p* = 0.304, refer to Additional file 1: Table S4).

### Quality of life

**Fig. 10:**
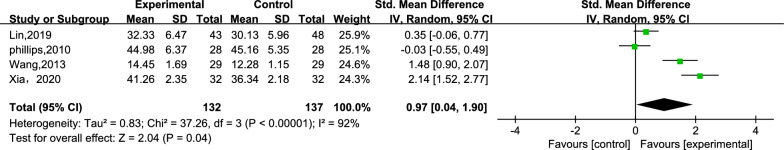
Meta-analysis of quality of life

**Fig. 11:**
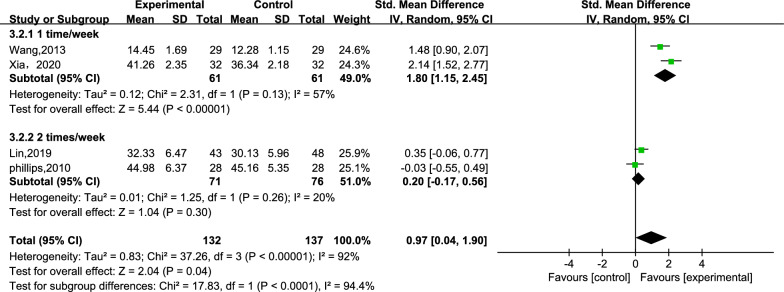
Subgroup analysis by intervention frequency of quality of life

Four studies examined the impact of the intervention on quality of life [27, 28, 30, 32]. In total, these studies included 269 subjects, with 132 subjects in the creative story therapy group and 137 subjects in the routine care group. The meta-analysis findings demonstrated that the creative story therapy group had significantly higher quality of life scores than the routine care group (SMD = 0.97, 95% CI 0.04, 1.90, *p* = 0.04, *I*^2^ = 92%, refer to Fig. 10). Given the high heterogeneity, a subgroup analysis was conducted based on the intervention frequency. The subgroups were categorized as “once in 1 week” and “twice a week” (refer to Fig. 11). The pooled results indicated a favorable outcome for the "Once in 1 week" subgroup, with reduced heterogeneity (SMD = 1.80, 95% CI 1.15, 2.45, *I*^2^ = 57%, *p* < 0.00001, 2 studies).

The sensitivity analysis revealed that the combined effect size was influenced by the removal of any literature source except for Phillips et al. (refer to Additional file 1: Table S2) [28]. Consequently, the pooled results should be interpreted with caution due to their instability.

### Social communication

**Fig. 12:**
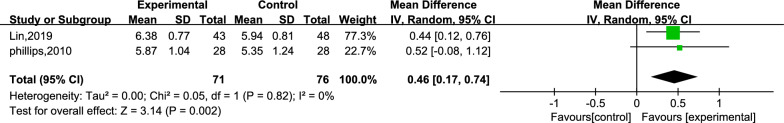
Meta-analysis of the social communication

Two studies examined the impact of the creative story therapy on social communication [27, 28]. These studies included a total of 147 subjects, with 71 subjects in the creative story therapy group and 76 subjects in the routine care group. The pooled results of the meta-analysis revealed that the creative story therapy group had significantly higher scores in social communication than the routine care group (MD = 0.46, 95% CI 0.17, 0.74, *p* < 0.00001, *I*^2^ = 0%, refer to Fig. 12).

### Basic needs communication

**Fig. 13:**
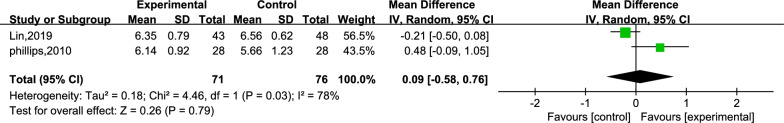
Meta-analysis of basic needs of communication

Two studies examined the impact of the creative story therapy on basic needs communication [27, 28]. These studies included a total of 147 subjects, with 71 subjects in the creative story therapy group and 76 subjects in the routine care group. There was no significant difference between the creative story therapy group and routine care group in basic needs communication (MD = 0.09, 95% CI − 0.58, 0.76, *p* < 0.00001, *I*^2^ = 78%, refer to Fig. 13).

## Discussion

This systematic review meta-analyzed the effects of creative story therapy combined with routine nursing on cognitive function, depression, quality of life, and communication in dementia patients. A total of 597 subjects participated in nine randomized controlled trials. The quality of the included studies was considered moderate overall. The research findings suggest that creative story therapy yields significant benefits in terms of cognitive function, reduction of depression, improvement of quality of life, and social communication compared to routine care in dementia patients. However, no significant difference was found in basic needs communication (Additional files 2, 3).

Creative story therapy combined with routine nursing appears to have a great potential in improving cognitive function of dementia patients. Among the seven studies evaluating cognitive function, six studies [25, 26, 29–32] reported positive effects, with effect sizes ranging from moderate to large (SMD ranged from 0.44 to 1.74) based on previous research [34]. Moreover, this meta-analysis suggests that an intervention duration of 4–8 weeks has a significant effect on improving cognitive function. Notably, creative story therapy exhibits superior effectiveness in improving cognitive function in AD compared to the studies that included patients with dementia. Creative story therapy, as a collective narrative project, stimulates the imagination of dementia patients, empowering them as active contributors rather than passive recipients during social activities. Through storytelling, patients can express themselves and the repeated review of activities aids in stimulating the cerebral cortex [35], thus improving memory function [36]. Previous studies have confirmed that the there is a relevance of the default mode network (DMN) functional connectivity for episodic memory function in AD. Zhao et al. [37] conducted creative story interventions on mild cognitive impairment (MCI) patients for 12 weeks, and found that creative story therapy could enhance the strength and range of brain function network connections in the elderly with MCI during the follow-up at six months later, and changes in brain network connections and activities helped to improve and maintain cognitive function in MCI patients. Similarly, Luo et al. [38] conducted a single-blind RCT on 73 MCI patients, demonstrating the same conclusion. Therefore, the mechanisms by which creative story therapy improves cognitive function in dementia may also be associated with the changes in DMN functional connectivity.

Creative story therapy combined with routine nursing may prevent depression in dementia patients [39]. Depression is an important factor that worsens the condition of dementia patients and reduces their quality of life [40]. Studies have shown that up to 50% of AD patients develop depressive symptoms or are diagnosed with depression during the course of their illness [39]. Creative story therapy is a social activity that is effective in reducing depression by increasing social activity in people with dementia [39]. In this process, dementia patients could actively express their outlook on life and values, and mutual communication can help alleviate negative emotions in patients and reduce depression. A systematic review showed that reminiscence therapy reminiscence, which takes a life story approach as the core intervention, can effectively improve the depressive state of AD patients, and they further noted that 30/35-min at least once a week over a period of 12 weeks is the optimal intervention duration [42]. Consistent with previous findings, our results suggested than an intervention duration of creative story therapy of more than 6 weeks has a better effect in reducing depression in patients.

Creative story therapy combined with routine nursing has the potential to enhance the quality of life of dementia patients, although conclusions are cautious. Among the four studies that reported on quality of life, two studies [30, 32] demonstrated significant positive effects (SMD ranged from 1.48 to 2.14) in improving the quality of life of dementia patients. Fay et al. provided creative story therapy to eight dementia patients over a period of 32 consecutive weeks during the COVID-19 pandemic, yielding a positive impact on crucial areas of quality of life, such as emotion, energy level, and cognitive function [21]. Furthermore, George et al. conducted a timeslips intervention involving 15 medical students, revealing that creative story therapy contributed to changing the students' attitudes toward dementia patients, improving their communication skills, and enhancing their interactions with patients [20]. This improvement in the quality of care ultimately promotes a better quality of life for patients. However, Houser et al. conducted a 6-week timeslips intervention with ten dementia patients and found no significant changes in emotional and behavioral symptoms [35]. Philip et al. did not find a significant change in quality of life, which may be due to its small sample size and complexity of sample source. In addition, his study pointed out that the benefits of the intervention were limited to the duration of the intervention, which hinted at the importance of extending the intervention period. Therefore, additional studies are warranted to further confirm the impact of creative story therapy on quality of life.

Creative story therapy combined with routine nursing may help improve social communication in dementia patients, although no significant difference was found in basic needs communication. Within this meta-analysis, only one of the two studies reporting on social communication in dementia patients demonstrated a positive effect, albeit with a small effect size (MD ranged from 0.44 to 0.52) [27]. Furthermore, two studies reported on basic needs communication in dementia patients, revealing no statistical difference in improvement between creative story therapy and routine care. This outcome could be attributed to the limited number and sample size of the included studies. Consequently, future research should prioritize conducting high-quality studies with larger sample sizes to confirm the impact of creative story therapy on social communication and basic needs communication.

This meta-analysis provides valuable insights into the effectiveness of creative story therapy, facilitating the further application of this therapeutic approach in dementia patients and emphasizing the importance of person-centered care philosophy in dementia management. However, our research has certain limitations. First, we solely searched for literature published in Chinese and English, excluding literature published in other languages. Second, the relatively small sample sizes in the included studies (with a minimum of 20 participants) are associated with the group intervention nature of creative story therapy. Thus, future research should prioritize conducting multicenter studies with larger sample sizes. The overall quality of the literature included in this review is also a factor influencing our conclusions. Limited descriptions of intervention implementation and blind evaluation methods in the included studies necessitate future high-quality research efforts, with a focus on implementing blinding measures to minimize bias.

## Conclusion

This comprehensive meta-analysis supported the effectiveness of creative story therapy combined with routine nursing in enhancing cognitive function and alleviating depression in individuals living with dementia. The findings from individual studies suggest promising outcomes in terms of improving quality of life and fostering social communication, although statistical significance was not observed in basic needs communication. Our research supports the importance of creative story therapy as a person-centered care intervention in nursing facilities and hospitals.

### Supplementary Information

**Additional file 1:** Sensitivity analysis and publication of biased results.

**Additional file 2:** Literature retrieval strategy and results.

**Additional file 3:** Original data of creative expression story therapy included in the literature.

## Data Availability

The datasets used or analyzed during the current study are available from the corresponding author on reasonable request.

## Abbreviations

PWD: People with dementia
PCC: Person-centered care
PRISMA: Preferred Reporting Items for Systematic Reviews and Meta-Analyses
RCTs: Randomized controlled trials
MD: Mean difference
CSDD: Cornell Scale for Depression in Dementia
