# Supplementary material for: Effectiveness of creative story therapy for dementia: a systematic review and meta-analysis
Source: Eur J Med Res. 2023 Sep 14;28:342. doi: 10.1186/s40001-023-01337-7 (PMC10500921; doi:10.1186/s40001-023-01337-7)
Supplement: Supplementary file 1 — Additional file 1: Sensitivity analysis and publication of biased results. [file 40001_2023_1337_MOESM1_ESM.docx]

**Table S1** Quality of included literature

| **First Author (Year)** | **Random sequence generation** | **Allocation concealment** | **Blinding of participants and personnel during the trial and adherence** | **Blinding of of outcome**  **assessment** | **Incomplete outcome data** | **Risk of bias in selection of the repor ted result** | **Other bias** | **Overall risk of bias Assessment** |
| --- | --- | --- | --- | --- | --- | --- | --- | --- |
| Phillips  (2010) | N | N | N | N | Z | Y | Y | High |
| Wang  (2013) | N | N | N | Y | Y | Y | Y | Some concerns |
| Lin  (2019) | Y | Y | Y | Y | Y | Y | Y | Low |
| Ye  (2019) | Y | Y | Y | Y | Y | Y | Y | Low |
| Cai  (2019) | Y | N | N | N | Y | Y | Y | Some concerns |
| Wang  (2020) | Y | N | Z | Z | Y | Y | Y | High |
| Xia  (2020) | N | N | Z | Z | Y | Y | Y | High |
| Shen  (2021) | Y | N | N | Y | Y | Y | Y | Some concerns |
| Fan  (2021) | Y | N | N | N | Y | Y | Y | Some concerns |

Z *–* high risk; Y – low risk;N –unclear

**Overall risk of bias assessment:**

**Low risk of bias:** The study is judged to be at **low risk of bias for all domains**for this result.

**Some concerns:** The study is judged to raise unclear in at least one domain for this result, but not to be at high risk of bias for any domain.

**High risk of bias:** The study is judged to be at **high risk of bias**in at least one domain for this result. Or: The study is judged to have **some concerns**for **multiple domains**in a way that substantially lowers confidence in the result.

**Table S2** Effect sizes.

| **Sensitivity Analysis** | **Heterogeneity test** | **Effect size：SMD/MD（95%CI）** |
| --- | --- | --- |
| **Cognitive functioning** |  |  |
| Previous result | H eterogeneity:Tau² = 0.25 Chi² = 30, df = 6 (P<0.00001); I² = 80% | 0.99 [0.57, 1.41] |
| Method 1： |  |  |
| Changing the analysis model | H eterogeneity: Chi² = 18.67, df = 13 (P<0.00001); I² = 80% | 0.98 [0.80, 1.17] |
| Method 2： |  |  |
| Cai,2019 excluded | H eterogeneity: Tau² = 0.26 Chi² = 26.88, df = 5 (P<0.00001); I² = 81% | 1.17 [0.62, 1.52] |
| Fan,2021 excluded | H eterogeneity:Tau² = 0.26 Chi² = 24.09, df = 5 (P = 0.0002); I² = 79% | 0.91 [0.45, 1.37] |
| Lin,2019 excluded | H eterogeneity:Tau² = 0.10 Chi² = 12.61, df = 5 (P = 0.03); I² = 60% | 1.15 [0.81, 1.48] |
| Shen,2021 excluded | H eterogeneity:Tau² = 0.20 Chi² = 20.7, df = 5 (P = 0.0009); I² = 76% | 0.87 [0.46, 1.28] |
| Wang,2013 excluded | H eterogeneity:Tau² = 0.31 Chi² = 29.99, df = 5 (P<0.00001); I² = 83% | 1.00 [0.51, 1.48] |
| Wang,2020 excluded | H eterogeneity:Tau² = 0.32 Chi² = 30.00, df = 5 (P<0.00001); I² = 83% | 0.99 [0.49, 1.49] |
| xia,2020 excluded | H eterogeneity:Tau² = 0.30 Chi² = 29.53, df = 5 (P<0.00001); I² = 83% | 0.96 [0.45, 1.45] |
| **CSDD** |  |  |
| Previous result | H eterogeneity: Tau² = 2.21 Chi² = 17.96, df = 4 (P = 0.001); I² = 78% | -1.71 [-3.27, -0.14] |
| Method 1： |  |  |
| Changing the analysis model | H eterogeneity: Chi² = 17.96, df = 4 (P = 0.001); I² = 78% | -2.22 [-2.28, -1.56] |
| Method 2： |  |  |
| Lin,2019 and phillips,2010 excluded | H eterogeneity: Chi² = 2.95, df = 2 (P = 0.23); I² = 32% | -3.21 [-4.06, -2.36] |
| **Quality of Life** |  |  |
| Previous result | H eterogeneity: Tau² = 0.83 Chi² = 37.26, df = 3 (P<0.00001); I² = 92% | 0.97 [0.04, 1.90] |
| Changing the analysis model |  |  |
| Cho (2018) | H eterogeneity: Chi² = 37.26, df = 3 (P<0.00001); I² = 92% | 0.79 [0.53, 1.05] |
| Method 2： |  |  |
| Lin,2019 excluded | H eterogeneity: Tau² = 1.22 Chi² = 30.29, df = 2 (P<0.00001); I² = 93% | 1.19 [-0.10, 2.48] |
| phillips,2010 excluded | H eterogeneity: Tau² = 0.85 Chi² = 24.85, df = 2 (P<0.00001); I² = 92% | 1.31 [0.22, 2.40] |
| Wang,2013 excluded | H eterogeneity: Tau² = 0.98 Chi² = 30.58, df = 2 (P<0.00001); I² = 93% | 0.81 [-0.36, 1.97] |
| xia,2020 excluded | H eterogeneity: Tau² = 0.44 Chi² = 15.21, df = 2 (P = 0.002); I² = 87% | 0.59 [-0.22, 1.39] |

**Table S3** Egger's test cognitive function

| Std_Eff | Coefficient | Std. err. | t | P>t | [95% conf. | interval] |
| --- | --- | --- | --- | --- | --- | --- |
|  |  |  |  |  |  |  |
| slope | .7106434 | 1.687852 | 0.42 | 0.691 | -3.628118 | 5.049405 |
| bias | 1.142553 | 6.754227 | 0.17 | 0.872 | -16.21974 | 18.50485 |

**Table S4** Egger's test of CSDD

| Std_Eff | Coefficient | Std. err. | t | P>t | [95% conf. | interval] |
| --- | --- | --- | --- | --- | --- | --- |
| slope | 4.231913 | 1.755565 | -2.41 | 0.095 | -9.818904 | 1.355079 |
| bias | 2.894735 | 2.338906 | 1.24 | 0.304 | -4.548707 | 10.33818 |
