# Supplementary material for: Effectiveness of creative story therapy for dementia: a systematic review and meta-analysis
Source: Eur J Med Res. 2023 Sep 14;28:342. doi: 10.1186/s40001-023-01337-7 (PMC10500921; doi:10.1186/s40001-023-01337-7)
Supplement: Supplementary file 2 — Additional file 2: Literature retrieval strategy and results. [file 40001_2023_1337_MOESM2_ESM.docx]

**The retrieval strategies and retrieval results of each database are shown in Tables 1-8**.

Table 1: PubMed

| No. | Content | Result |
| --- | --- | --- |
| #1 | "Alzheimer Disease"[MeSH Terms] OR Dementia[MeSH Terms] | 202,539 |
| #2 | Alzheimer Type Senile Dementia[Title/Abstract] OR Alzheimer Dementia[Title/Abstract] OR Alzheimer Dementias[Title/Abstract] OR Dementia, Alzheimer[Title/Abstract] OR Alzheimer Disease[Title/Abstract] OR Dementia, Senile[Title/Abstract] OR Senile Dementia[Title/Abstract] OR Dementia, Alzheimer Type[Title/Abstract] OR Alzheimer Type Dementia[Title/Abstract] OR Senile Dementia, Alzheimer Type[Title/Abstract] | 25,273 |
| #3 | #1 OR #2 | 209,503 |
| #4 | "creative"[Title/Abstract] OR "creative expression"[Title/Abstract] OR "timeslips"[Title/Abstract] | 16,791 |
| #5 | **#3 AND #4** | 218 |

Table 2: Embase

| No. | Content | Result |
| --- | --- | --- |
| #1 | 'alzheimer disease'/exp OR Dementia /exp | 436,994 |
| #2 | ''alzheimer type senile dementia':ab,ti OR 'alzheimer dementia':ab,ti OR 'alzheimer dementias':ab,ti OR 'dementia, alzheimer':ab,ti OR 'alzheimer disease':ab,ti OR 'dementia, senile':ab,ti OR 'senile dementia':ab,ti OR 'dementia, alzheimer type':ab,ti OR 'alzheimer type dementia':ab,ti OR 'senile dementia, alzheimer type':ab,ti OR 'vascular dementia':ab,ti | 316,203 |
| #3 | #1 OR #2 | 429,180 |
| #4 | **creative:ab,ti OR 'creative expression':ab,ti OR 'timeslips':ab,ti** | 23,238 |
| #5 | **#3 AND #4** | 20 |

Table 3: Web of science

| No. | Content | Result |
| --- | --- | --- |
| #1 | TS=(Alzheimer Disease OR Dementia) | 288100 |
| #2 | AB=(Alzheimer Type Senile Dementia OR Alzheimer Dementia OR Alzheimer Dementias OR Dementia, Alzheimer OR Alzheimer Disease OR Dementia, Senile OR Senile Dementia OR Dementia, Alzheimer Type OR Alzheimer Type Dementia OR Senile Dementia, Alzheimer Type OR Vascular dementia) | 139,646 |
| #3 | #2 OR #3 | 296,508 |
| #4 | **AB=(**creative OR creative expression OR timeslips) | 72,463 |
| #5 | **#3 AND #4** | 390 |

Table 4: Cochrance

| No. | Content | Result |
| --- | --- | --- |
| #1 | (Alzheimer Type Senile Dementia OR Alzheimer Dementia OR Alzheimer Dementias OR Dementia, Alzheimer OR Alzheimer Disease OR Dementia, Senile OR Senile Dementia OR Dementia, Alzheimer Type OR Alzheimer Type Dementia OR Senile Dementia, Alzheimer Type):ti,ab,kw | 13,357 |
| #2 | (creative OR creative expression OR timeslips):ti,ab,kw | 1657 |
| #3 | **#1 AND #2** | 22 |

Table 5: CNKI

| No. | Content | Result |
| --- | --- | --- |
| #1 | (主题=老年痴呆 + 老年性痴呆 + AD + 阿尔茨海默病 + 阿尔兹海默病) | 414,979 |
| #2 | (篇关摘=创造性故事疗法 + 生活故事 + 创造性表达 + timeslips) | 3404 |
| #3 | #1 AND #2 | 20 |

Table 6: WANGFANG

| No. | Content | Result |
| --- | --- | --- |
| #1 | 题名或关键词:(老年痴呆 or老年性痴呆or AD or 阿尔兹海默病 or 阿尔茨海默病) | 24,569 |
| #2 | 题名或关键词:(创造性故事疗法or 生活故事 or 创造性表达 or timeslips) | 22 |
| #3 | #1 AND #2 | 14 |

Table 7: VIP

| No. | Content | Result |
| --- | --- | --- |
| #1 | (主题=老年痴呆 + 老年性痴呆 + AD + 阿尔茨海默病 + 阿尔兹海默病) | 82,203 |
| #2 | (摘要=创造性故事疗法 + 生活故事 + 创造性表达 + timeslips） | 28,579 |
| #3 | #1 AND #2 | 29 |

Table 8: CBM

| No. | Content | Result |
| --- | --- | --- |
| #1 | 老年痴呆"[常用字段:智能] OR老年性痴呆[常用字段:智能] OR "阿尔茨海默病"[常用字段:智能] OR "阿尔兹海默病"[常用字段:智能] OR "痴呆"[常用字段:智能] OR "AD"[常用字段:智能] | 233,907 |
| #2 | "创造性故事疗法"[常用字段:智能] OR "生活故事"[常用字段:智能] OR "创造性表达"[常用字段:智能] OR "timeslips"[常用字段:智能] | 67 |
| #3 | #1 AND #2 | 18 |
